# In silico prediction of Ebola Zaire GP_1,2_ immuno-dominant epitopes for the Balb/c mouse

**DOI:** 10.1186/s12865-015-0126-8

**Published:** 2015-10-06

**Authors:** Debargh K. Dutta, Kelly Rhodes, Steven C. Wood

**Affiliations:** Division of Biology, Chemistry and Materials Science, Center for Devices and Radiological Health, FDA, Silver Spring, MD 20993 USA; Department of Medicine, USUHS, 4301 Jones Bridge road, Bethesda, MD 20814 USA; University of Maryland, College Park, MD 20742 USA

**Keywords:** Ebola, GP_1,2_, Immunodominant epitopes, CTL, Tetramer

## Abstract

**Background:**

Ebola is a Filovirus (FV) that induces a highly communicable and deadly hemorrhagic fever. Currently, there are no approved vaccines to treat FV infections. Protection from FV infection requires cell mediated and humoral immunity. Glycoprotein GP_1,2_ Fc Zaire, a recombinant FV human Fc fusion protein, has been shown to confer protection against mouse adapted Zaire Ebola virus. The present studies are focused upon identifying immunodominant epitopes using *in silico* methods and developing tetramers as a diagnostic reagent to detect cell mediated immune responses to GP_1,2_ Fc.

**Methods:**

The GP_1,2_ Ebola Zaire sequence from the 1976 outbreak was analyzed by both BIMAS and SYFPEITHI algorithms to identify potential immuno-dominant epitopes. Several peptides were synthesized and screened in flow-based MHC stability studies. Three candidate peptides, P8, P9 and P10, were identified and, following immunization in Balb/c mice, all three peptides induced IFN-γ as detected by ELISpot and intracellular staining.

**Results:**

Significantly, P8, P9 and P10 generated robust cytotoxic T-cell responses (CTL) as determined by a flow cytometry-based Caspase assay. Antigen specific cells were also detected, using tetramers. Both P9 and P10 have sequence homology with highly conserved regions of several strains of FV.

**Conclusions:**

In sum, three immunodominant sequences of the Ebola GP_1,2_ have been identified using *in silico* methods that may confer protection against mouse adapted Ebola Zaire. The development of tetramer reagents will provide unique insight into the potency and durability of medical countermeasure vaccines for known bioterrorism threat agents in preclinical models.

## Background

Filoviruses (FV) are single-stranded negative sense RNA viruses that infect humans and primates. FV quickly obtunds the immune system and dysregulates coagulation [1]. The mortality rate can be up to 90 % and is species-dependent. FV are Category A bioterrorism agents, and there are currently no licensed vaccines or treatments. FV induce a myriad of cellular effects that quickly overwhelm the immune system, which results in compromised FV antigen processing and presentation capabilities [2], depleted T-cells and poor cytotoxic T-cell (CTL) response [3, 4] and abrogated protective antibody production [3, 5].

The Ebola GP_1,2_ glycoprotein is responsible for viral binding and entry. It is a trimer with a chalice-like form [6]. The receptor binding region is inside the chalice and the exterior is highly glycosylated. The receptors binding domains and heptad repeats in the base are highly conserved sequences among the FV species. The ability to generate a cellular T-cell response contributes to surviving the disease. The role of T-cells in controlling Ebola and Marburg infections has been recognized [3, 4, 7]. In mice, CD8^+^ T-cell depletion and perforin knockouts studies using FV have shown the need for CTL to survive infection [8]. However, the CTL responses to FV in non-human primates have not been well characterized given the technical challenges of working in a BSL-4 environment.

Several FV vaccine constructs have been demonstrated to be protective in mice, guinea-pigs and non-human primates (NHP). Irradiated Ebola virus encapsulated in liposomes containing lipid A elicited GP_1,2_ specific CTL response [9] and attenuated Venezuelan equine encephalitis virus replicons expressing GP_1,2_ [10] showed protective responses in mice. An improved vaccination strategy using DNA plasmids expressing mixtures of different GP_1,2_ along with nucleoprotein (NP) and further boosting with recombinant adenovirus expressing GP_1,2_ proved effective in eliciting protection against live Ebola virus challenge in cynomolgus macaques [11]. Vesicular stomatitis virus based vaccines encoding GP_1,2_ protected NHP from Bundibugyo Ebola virus [12]. Respiratory tract immunization of NHP with a Newcastle disease virus-vectored vaccine showed protective response against Ebola virus challenge [13]. Live attenuated rabies virus vaccine expressing GP_1,2_ has been proposed for its safe use for vaccination in humans and wildlife [14]. Also, the recombinant protein, Ebola Zaire GP_1,2_ Fc, protected mice from lethal mouse adapted Ebola Zaire challenge [15].

Identifying immunodominant epitopes provides critical insight into the mechanism of protection to FV. Immunodominant peptides of EBOV GP_1,2_ [16–19] and Lassa GP_1,2_ and Ebola nucleoprotein have been determined [20, 21]. Importantly, MHC tetramer technology has simplified the study of antigen-specific CTLs to determine the functional sensitivities of T-cell populations [22]. Tetramers are fluorochrome-conjugated peptide–major histocompatibility complex (pMHC) multimers that are used in the study of antigen-specific T-cells by enabling their visualization, enumeration, phenotypic characterization and have been extensively used to track T-cells *ex-vivo* following vaccination or during viral infection [23]. As a result, tetramers can be used as surrogate markers of cell mediated immunity. On the other hand, B-cell mediated responses, especially antibodies specific to GP_1,2_ co-related with protection in NHPs and mice [24, 25]. The postexposure antibody treatments demonstrated to protect NHPs from filovirus infection [26]. In sum, development of effective Ebola vaccine would require both protective CTLs as well as a robust antibody response to ensure survival to lethal Ebola challenge.

In the present study we identified immunodominant peptides of the GP_1,2_ from Ebola Zaire in Balb/C mice for the K^D^ MHC using *in silico* algorithms. The objective was to generate tetramers that can be used to follow CTL. Peptide candidates were screened and three peptides stabilized the K^D^ MHC: P8, EYLFEVDNL, amino acids (aa) 231–240; P9, LFLRATTEL, aa 571–579; and P10, LYDRLASTV, aa 161–169. Splenocytes from peptide immunized mice generated IFN-γ upon *in vitro* re-stimulation and induced apoptosis in peptide sensitized targets. Tetramers for all three peptides were generated that detected antigen specific T-cells following immunization. Significantly, two of the three epitopes are conserved within FV which suggests that tetramer reagents will be useful in following cell mediated responses to Zaire vaccine candidates such as GP_1,2_ recombinant proteins, GP_1,2_ expressing virus like particles, recombinant VSV, Adenoviruses and GP_1,2_ DNA vaccines.

## Results

### In silico predictions of GP_1,2_ immuno-dominant epitopes

The GP_1,2_ sequence of Ebola Zaire was analyzed using both BIMAS and SYFPEITHI algorithms to identify peptides that may possess high binding affinity to K^D^ MHC. Three peptides scored better than 150 for BIMAS and 20 for SYFPEITHI, and these are presented in Table 1. These were P8, EYLFEVDNL amino acids (AA) 231–240 [16]; P9, LFLRATTEL AA 571–579; and P10, LYDRLASTV AA 161–169 [17]. These peptides were synthesized for further characterization. This is the first report of evaluation of immunological response analysis for peptide P9. The peptide P9 (LFLRATTEL), was identified in 2009 by Kent et al., (Kent J. Weinhold, Georgia D. Tomaras, Yongting Cai, Kelly Plonk, Scott Pruitt, Smita K.Nair) and was submitted to Immune Epitope Database and analysis Resource and can be found at the following link, http://www.iedb.org/epId/91636. Peptides P8 and P10 were previously reported by Dowling et al. [16] and Warfield et al. [17] respectively.

**Table 1:** *In silico* predictions of Ebola Zaire GP_1,2_ epitopes

Protein sequence	Start position	Peptide	BIMAS	SYFPEITHI
			Score	Score
Ebola Zaire GP	231	EYLFEVDNL	4000	23
676 AA		(P8)		
	571	LFLRATTEL	1920	25
		(P9)		
NCBI Accession number Q05320.1	161	LYDRLASTV	600	22
		(P10)		

GP_1,2_ from Ebola Zaire was analyzed both by BIMAS and SYFPEITHI. Nonomer peptides that scored greater or equal to 150 on BIMAS and 22 on SYFPEITHI were subjected to further evaluation

### MHC stability studies using RMAS K^D^ cells

**Fig. 1:**
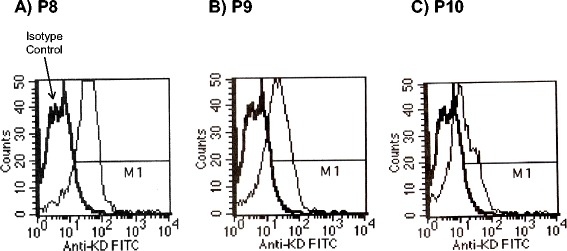
Stabilization of K^D^ expression IN K^D^ RMAS cells by Ebola GP_1,2_ peptides. GP_1,2_ Ebola Zaire, NCBI accession number AAB81004.1, was analyzed by both BIMAS and SYFPEITHI. The GP_1,2_ protein sequence was divided into segments of nine amino acid that overlapped by eight amino acids. Peptides that scored better than >150 in BIMAS and >24 in SYFPEITHI were selected for further study. BD FACS SCAN was used to assess peptide stabilization of MHC expressed on K^D^ RMAS cells. The heavy line represents isotype control. The results are representative of three independent experiments

The peptides identified above using the BIMAS and SYFPEITHI algorithms were screened using a MHC stability assay. Peptides were incubated with TAP deficient RMAS cells that expressed the Class I molecule, K^D^. Following an overnight incubation, K^D^ was assessed in a flow-based assay. The MFI for the three peptides is shown in Fig. 1. All three peptides induced a significant increase in the MFI, as compared to the isotype control (6 ± 0). P8 was 45 ± 4, P9 was 19 ± 5, and P10 was 17.6 ± 0.5.

**Fig. 2:**
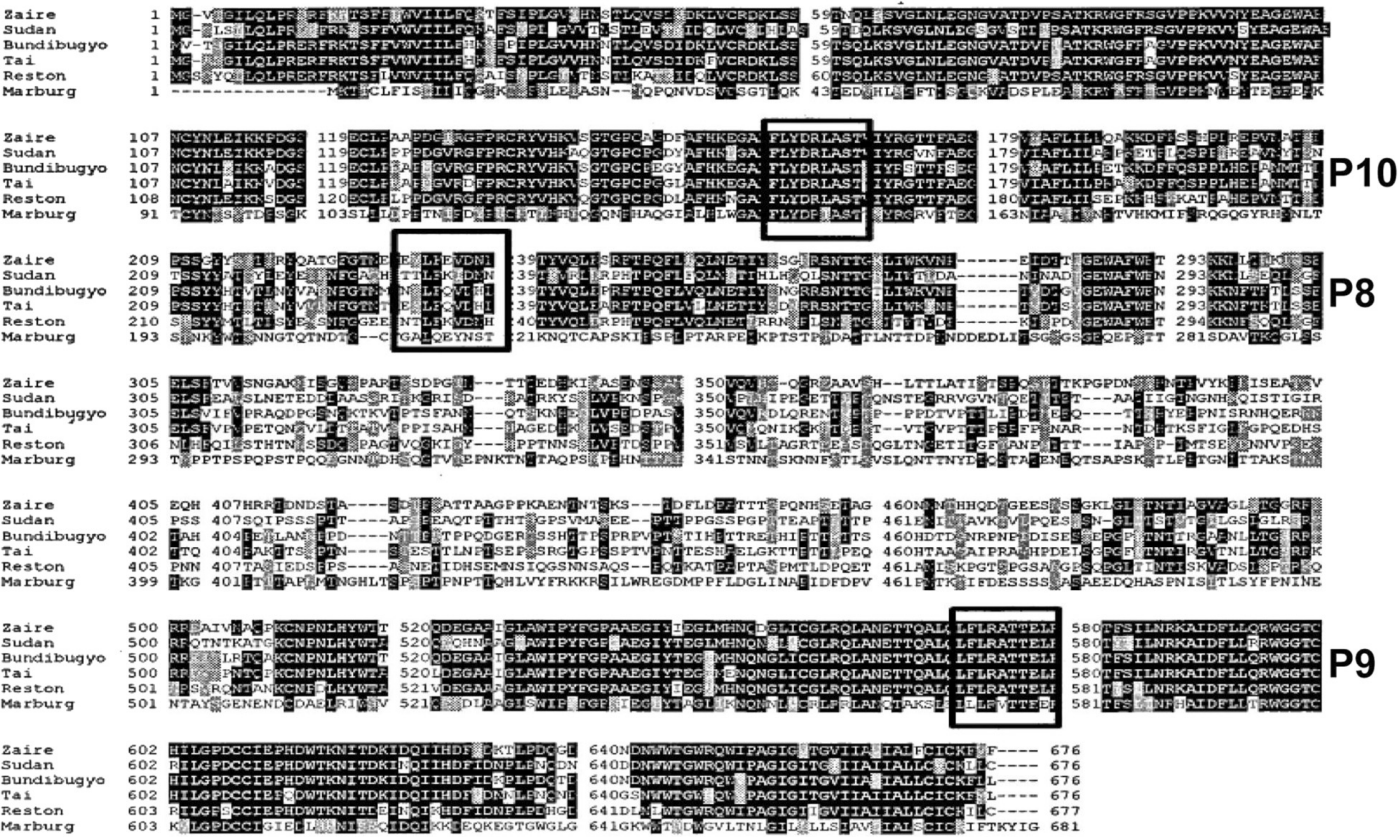
Sequence alignment of GP_1,2_ from Ebola Zaire (AAA96744.1), Sudan virus (AAP88031.1), Bundibugyo (YP_003815435), Tai Forest (YP_003815426.1), Reston (Q66799.1), and Marburg Lake Victoria (P35254.1). Clustal W alignments were performed and highlights were added via Boxshade. Amino acids that are identical are shaded black while chemically similar amino acids are grey. The location of peptides P8, P9 and P10 are indicated by the boxes. P8 is in a highly variable region while P9 and P10 are located in highly conserved regions for cytosolic entry and cellular recognition, respectively

These results suggest that P8, P9 and P10 can stabilize the K^D^ MHC. P8 originates from the highly variable and glycosylated region of GP_1,2_. P9 is located in the conserved region of the tail that is responsible for injecting the virus into the cytoplasm. P10 is found in the conserved region that mediates GP_1,2_ receptor binding to cellular receptors. The sites of these peptides are shown in Fig. 2 and are overlaid on the GP_1,2_ sequences for Bundibugyo, Tai Forest, Zaire, Reston, Sudan and Marburg viruses.

### ELISpot analysis of peptides

**Fig. 3:**
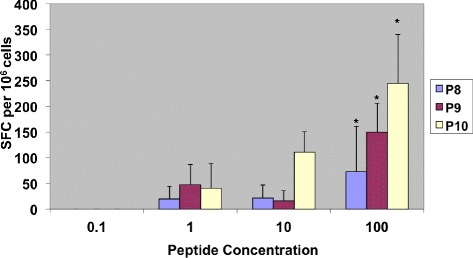
IFN-γ Elispot responses by splenocytes were evaluated following immunization with Ebola Zaire GP_1,2_ peptides. Mice were immunized and boosted 7 days after initial immunization. Splenocytes were re-stimulated *in vitro* with peptide overnight. The mean IFN- γ Elispots/10^6^ splenocytes +/− the S.E. is presented. The results are representative of three independent experiments. Asterisks indicate statistically significant IFN- γ increase in peptide-exposed cells at 100 mm concentration compared with those cells treated with 0.1 mm peptide (* = *p* <0.05)

An ELISpot for IFN-γ was used to assess the immunodominant potential of the peptides identified in the K^D^ stability studies. To assess immunogenic potential, mice were immunized with P8, P9 or P10 and an IFN-γ ELISpot was performed. As shown in Fig. 3, the P8 ELISpot response was less robust when compared to P9 and P10. For P10, the ELISpot response was concentration-dependent and the maximum response for the peptide was 100 μM.

### Intracellular staining for IFN-γ

**Fig. 4:**
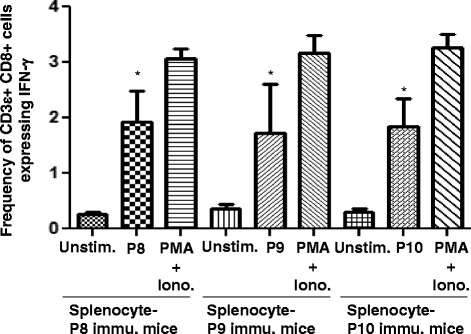
Induction of intracellular IFN- γ production following immunization with P8, P9 and P10. Splenocytes from peptide immunized animals were cultured for 24 h *in vitro* with respective peptide. Cells were collected, washed, stained with CD3ε PerCP and anti-CD 8 FITC and fixed. The cells were further stained with anti-IFN-γ antibody and were analyzed using BD FACS Canto. The results are represented as mean +/− S.E. of three independent experiments. Asterisks indicate statistically significant IFN-γ increase in peptide-exposed cells compared with those untreated cells (* = *p* <0.05)

To clarify the cellular identity of IFN-γ secretions, intracellular staining (ICS) was performed using splenocytes from peptide immunized mice. A robust signal for IFN-γ was seen for all three peptides that were predominately in the CD8^+^ T-cells as shown in Fig. 4. In sum, the three peptides induced IFN-γ expression in CD8^+^ T-cells, which was around 2 % as compared to the control of 0.25 %.

### Tetramer analysis

**Fig. 5:**
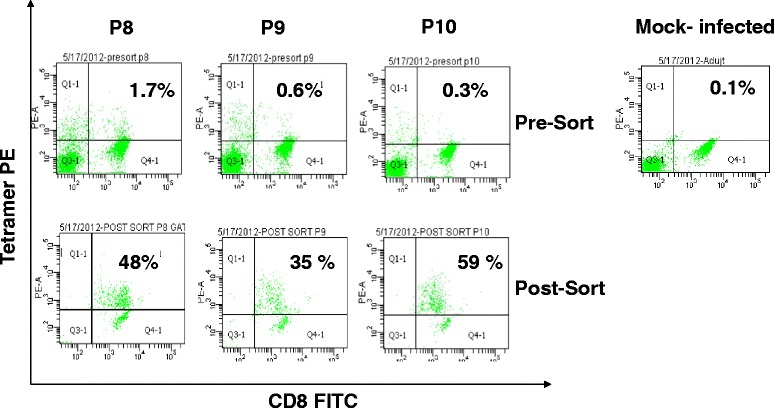
Tetramer staining of immunized splencocytes. Splenocytes from peptide immunized animals were stained with K^D^ tetramers prepared with P8, P9, P10 or Adjuvant (mock-infected). The cells were counter stained with CD8 FITC and CD3 PerCP, then fixed. The stained cells were analyzed in a FACS ARIA. Tetramer positive cells were sorted and re-analyzed. Pre-sort and Post sorted tetramer positive cells are shown in figure. The results are representative of three independent experiments

To follow the development of CTL cells, Tetramer reagents for K^D^ MHC were generated for P8, P9 and P10. Mice were immunized with peptides as described above and splenocytes were prepared. Splenocytes were stained with tetramer and counterstained with CD3 PerCP and CD8 FITC. The results are shown in Fig. 5. In the CD8^+^ T-cell gated population, 1.7 % of cells were positive for P8 tetramer, 0.6 % of cells were positive for P9 tetramer, and 0.3 % of cells were positive for P10 tetramer. Clearly, antigen specific cells can be induced by P8, P9 and P10 and tetramers bound to splenocytes can be detected and amplified by sorting. Next, cells were sorted on the basis of tetramer binding. The post-sort results are also shown in Fig. 5. The frequency of tetramer positive cells after sorting was increased to 48 % (28 fold), 35 % (58 fold) and 59 % (196 fold) for P8, P9 and P10, respectively.

### CTL analysis

**Fig. 6:**
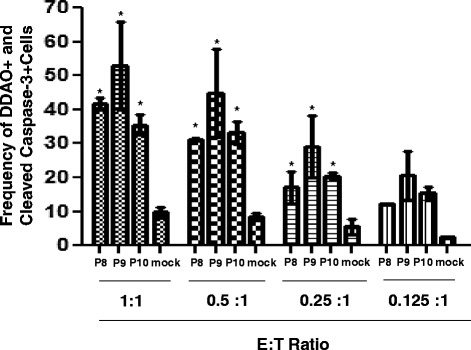
CTL response induced by peptides P8, P9, and P10. Splenocytes from immunized animals were cultured for 5 days *in vitro* with peptide, collected and counted. In parallel, K^D^ cells were incubated overnight with P8, P9, P10 or adjuvant (mock control), washed and then labeled with DDAO cell tracker. Effector and target cells were mixed and incubated for 4 h in the indicated ratios and fixed. The cells were stained with anti-cleaved Caspase-3 PE and analyzed by flow cytometry using a FACS Canto. The mean percentage of Caspase positive K^D^ cells +/− the SE is presented. The results are representative of three independent experiments. Asterisks indicate statistically significant increase in Caspase -3 positive cells compared with those Caspase positive cells in mock (* = *p* <0.05)

Finally, the ability of P8, P9 and P10 to generate functional CTL was examined. A flow based assay was used to follow Caspase 3 generation in K^D^ targets. Splenocytes from peptide immunized mice were re-stimulated *in vitro* with the corresponding peptides. K^D^ targets were incubated overnight with peptides. The effector cells and targets were mixed and the Caspase 3 expression was followed by flow cytometry as shown in Fig. 6. For all three peptides, Caspase generation was seen that was dependent upon the E: T ratio. With all three peptides, significant killing was seen at E: T ratios of 1:1, 0.5:1, 0.25:1 and 0.125:1. The robust lysis implies that P8, P9 and P10 may function *in vivo* as immunodominant epitopes.

## Discussion

In the current study, several hundred Ebola GP_1,2_ Zaire sequences were examined using *in silico* techniques. Nanomers that scored higher than 22 on SYFPEITHI or 150 on BIMAS are shown in Table 1. The SYFPEITHI algorithm provides a score based upon the pattern of amino acid sequences that preferentially bind to the MHC. The BIMAS score is based on binding affinities of single amino acids of the peptide, with each amino acid weighted the same, thereby providing an overall affinity to MHC. Following their synthesis, MHC stability was examined using K^D^ expressing RMAS cells. The peptides were P8, EYLFEVDNL aa 231–240; P9, LFLRATTEL, aa 571–580 and P10, LYDRLASTV aa 161–170. P8 and P10 are from regions that are highly conserved across species Zaire Mayinga, Zaire Kikwit, Cote-d-Ivoire, Sudan Boniface, Sudan Gulu, Reston Virginia and Marburg Popp [6]. P8 [18, 19] and P10 [17–19] have been previously reported as CTL epitopes in Zaire EBOV GP_1,2_; however we have performed immunological analysis of a immune-dominant P9 peptide, which has been submitted by Kent et al. to http://www.iedb.org/epId/91636. As shown in Fig. 1, all three stabilized expression of K^D^ MHC, which is crucial for immunodominant epitopes.

ELISpot identifies cells secreting cytokines and has a limit of detection as low as 1:10^6^ [27, 28]. The ELISpot also provides a convenient means of estimating the frequencies of IFN-γ producing cells [29]. Using a rabies vaccine expressing Ebola virus glycoprotein, a strong ELISpot response was seen in mice using peptide pools of GP_1,2_ [14]. In the study, the peptides P8, P9 and P10 induced IFN-γ release. However as shown in Fig. 2, in contrast to the K^D^ stability studies, P10 elicited the greatest response followed by P9 and P8.

As shown in Fig. 3, all three peptides induced IFN-γ in CD8^+^ T-cells, implying that these peptides may provide significant protection against Ebola. Lassa GP_1,2_ peptide also elicited a IFN-γ signal in HLA-A2.1 transgenic mice [20]. The ICS results were highly consistent with the ELISpot data.

Class I tetramers permit the rapid assessment of antigen specific CD8^+^ T-cells which is essential in monitoring immune responses to infections as well as vaccine development [23]. Tetramers can often detect antigen specific T-cells that are of low abundance and undetectable by functional studies such as ELISpot and ICS [30–33]. Tetramers were developed for nucleoprotein epitopes of Ebola Zaire [8]. In this study, peptides P8, P9 and P10 were used to generate Class I Tetramers (synthesized at NIAID). When mice were immunized with the peptides, a specific staining for each tetramer was observed as shown in Fig. 4. Furthermore, we sorted the cells based upon tetramer staining which greatly amplified the detection of epitope specific CD8^+^ T-cell population. This demonstrates the utility of the tetramer reagents that will greatly assist in the ongoing studies using GP_1,2_ from Ebola Zaire.

We have used a non-radioactive flow-based assay to detect apoptosis in peptide sensitized, DDAO labeled K^D^ RMAS cells [34]. Most importantly, the flow-based Caspase 3 generation assay is 10–50 times more sensitive than the chromium release assay [34]. As shown in Fig. 5, we noted Caspase 3 in DDAO labeled targets at the low Effector to Target ratio of 1:1. The Caspase generation is a function of effector cell number and was reduced linearly for all three peptides as the number of effectors decreased. The Caspase-3 expression shown in Fig. 6 and the IFN-γ expression observed in ICS staining as seen in Fig. 4 were comparable and support each other. These results suggest that the peptides may play a significant role during vaccination and protection of Ebola infection. The results from the present study are consistent with earlier studies [9]. In Rao et al., the peptides P1 and P2 were identified using classical CTL assays to Ebola Zaire. Both P1 and P2 were assessed using liposomes containing Ebola Zaire, though the E:T ratio used by Rao et al. was much higher than used in the present experiments. CTL generated by peptides have also been demonstrated for Ebola Zaire NP [21] and Lassa GP_1,2_ peptides [20] using a Chromium release assay.

**Fig. 7:**
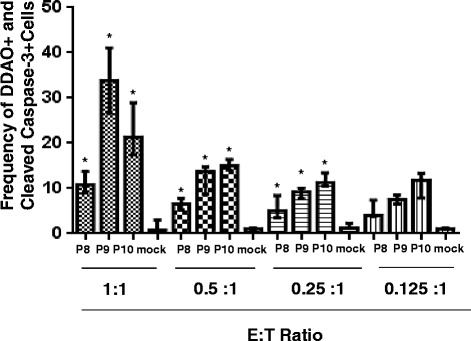
CTL response induced by GP_1,2_ immunization. Splenocytes from immunized animals were cultured for 5 days *in vitro* with P8, P9 and P10 peptides, for the expansion of peptide specific CTLs, that were collected and counted. In parallel, K^D^ cells were incubated overnight with P8, P9, P10 or adjuvant (mock control), washed and then labeled with DDAO cell tracker. Effector and target cells were mixed and incubated for 4 h in the indicated ratios and fixed. The cells were then stained with anti-cleaved Caspase-3 PE and analyzed by flow cytometers, using a FACS Canto. The mean percentage of Caspase positive K^D^ cells +/− the SE is presented. The results are representative of three independent experiments. Asterisks indicate statistically significant increase in Caspase -3 positive cells compared with those Caspase-3 positive cells in mock (* = *p* < 0.05)

**Fig. 8:**
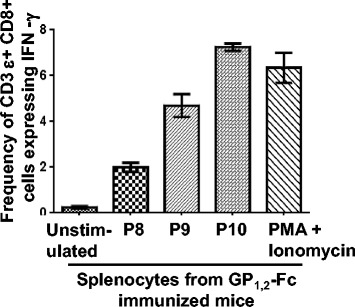
Induction of intracellular IFN-γ production following immunization with GP_1,2_-Fc. Splenocytes from GP_1,2_-Fc immunized animals were cultured for 24 h *in vitro* with P8, P9 and P10 peptides. Cells were collected, washed, stained with CD3ε PerCP and anti-CD 8 FITC and fixed. The cells were further stained with anti-IFN-γ antibody and were analyzed using BD FACS Canto. The results are represented as mean +/− S.E. of two independent experiments

It has been shown that GP_1,2_ can confer protection against mouse adapted Ebola [15]. It would be of significant interest to determine the immunogenicity of P8, P9 and P10, the immunodominant epitopes in GP_1,2_. Taking a step further, we have immunized Balb/c mice with GP_1,2_, the protein responsible for both cell mediated and humoral immunity. As shown in Fig. 7, all the peptides in our study specifically induce Caspase-3 expression (killing) in DDAO-labeled target cells. Fig. 8 shows induction of IFN-γ in CD8^+^ T-cells, when stimulated with peptides. This indicates that immunizing with an Ebola virus Glycoprotein, in a whole protein form, can potentially promote vaccine induced protective immunity. This experiment further confirms the immune-dominant potential of these peptides. Recently, a recombinant Vesicular Stomatitis Virus based vaccine, expressing Zaire-GP_1,2_ was found to be 100 % successful in phase I trail in Africa. Hence, identifying immune-dominant peptide specific protective CTLs in these protected vaccinees, using tetramer technology for human CD8^+^ T-cells, would aid in development of peptide-based vaccines against the deadly disease. Additionally, we would also like to extend our studies to explore the cell-mediated responses in transgenic mice expressing mamu-A-01 transgene, a Rhesus HLA to Ebola GP_1,2_. The challenge is in extending promising rodent studies to non-human primates [35].

## Conclusions

In this paper, we have identified three immune-dominant K^D^ epitopes of GP_1,2_ using *in silico* methods and evaluated their immunogenicity through *in vitro* and *in vivo* assays. Furthermore, we developed tetramers, which detect Ebola specific T-cells. These tetramer reagents will significantly contribute in evaluating cell-mediated responses to infection and also in determining the efficacy of prospective vaccine candidates in the field of Ebola vaccine research.

## Methods

### *In silico* epitope prediction

The amino acid sequence for the Ebola Zaire GP_1,2_ Mayinga 1976 strain, (NCBI accession number: AAB81004), was used in these experiments. The sequence, 676 aa long, was analyzed first using the BIMAS algorithm (http://www-bimas.cit.nih.gov/) followed by the SYFPEITHI algorithm (http://www.syfpeithi.de) for the K^D^ MHC of Balb/c [Parker, 1994 13 /id]. The following Filoviruses were used for sequence alignment of GP_1,2_, and their NCBI accession numbers are indicated: Zaire Ebola virus (AAA96744.1); Sudan Ebola virus (AAP88031.1); Ebola Bundibugyo (YP_003815435); Tai Forest (YP_003815426.1); Reston Ebola virus (Q66799.1) and Lake Victoria Marburgvirus (P35254.1).

### Peptides

Peptides were synthesized by New England Peptide (Gardner, MA), using standard methodology. Peptides were >95 % pure. They were re-constituted in de-ionized water and kept frozen at −20 °C until use. All peptides were diluted in culture media: RPMI-1640 (Gibco, Green Island, NY) containing 10 % (v/v) heat-inactivated fetal bovine serum (FBS), 1 % (v/v) antibiotic-antimycotic solution (Gibco) and 2 mM glutamine (Gibco).

### Mice

Balb/c female mice, 4–6 weeks of age, were obtained from the National Cancer Institute, Frederick, MD. Upon receipt, the mice were held for 7 days for acclimation in an Association for Assessment and Accreditation of Laboratory Animal Care International -approved facility with ad libitum access to food and water. All experiments were performed with the approval of the Food and Drug Administration, Center for Drug and Radiological Health Institutional Animal Care and Use Committee.

### Immunization

Mice were immunized intra-peritoneally with 100 μg of peptide and 140 μg of Hepatitis B virus helper peptide, TPPAYRPPNAPIL in 100 μl of Seppic Montanide (Fairfield, NJ). Initially, the peptides were diluted in sterile PBS and mixed with an equal volume of Seppic Montanide. Mice were boosted with equivalent dose on day 7. One week later, the mice were sacrificed with CO_2_ and their spleens were harvested. The spleens were placed in pre-warmed Hank’s Balanced Salt Solution (HBSS) and processed to single cell suspensions. The erythrocytes were lysed with Ammonium Chloride potassium lysing buffer (Gibco) and the cells were washed once and re-suspended in media. For GP_1,2_ immunization, 100 μg GP_1,2_ -Fc (Kindly, provided by Dr. Krishnamurthy Konduru, CBER/FDA) and 140 μg of Hepatitis B virus helper peptide, TPPAYRPPNAPIL in 100 μl of Seppic Montanide. Mice were boosted with equivalent dose on day 10. Ten days later, the mice were sacrificed with CO_2_ and their spleens were harvested. Spleens were then processed to single cell suspension as described above.

### ELISpot

The frequency of interferon-γ (IFN-γ) producing cells was measured by ELISpot. Briefly, the Immobilon®-P polyvinylidene fluoride plates (Millipore, Billerica, MA) were treated with 15 μl 33 % ethanol (Pharmco, Shelbyville, KY) for 1 min, and washed 5 times with sterile H_2_O. These plates were coated with 0.1 M carbonate Buffer (Sigma) containing anti-mouse IFN-γ (Mabtech, Mariemont, OH), 15 μg/ml and refrigerated overnight at 4 °C. The plate was washed 3 times in sterile PBS. The plate was incubated with blocking buffer using 1 % Bovine Serum Albumin in HBSS for 2 h at 37 °C in culture. 10^5^ splenocytes per well were plated within 1 h of harvest from peptide immunized mice and incubated with selected dilutions of peptide in six replicate wells. PMA and ionomycin treated splenocytes were used as a positive control in all experiments. Following an overnight incubation at 37 °C, the media was removed and the plate was incubated with 200 μl wash buffer (PBS containing 0.01 % Tween-20, Sigma) for 10 min at RT. Next, the plate was washed three times with wash buffer for 1 min at RT. The biotinylated detecting antibody (Mabtech) diluted 1:1000 in freshly made and filtered 1 % BSA/PBS was added and the plate was incubated for 2 h at RT. The plate was washed 3 times with wash buffer and twice with PBS. Next, streptavidin HRP (Jackson Immuno Research, West Grove, PA) diluted 1:500 in PBS containing 0.5 % FCS in PBS was added and the plate was incubated for 1 h at RT. Following washing, 50 μl TMB substrate (Mabtech) was added per well. Washing with deionized water stopped the reaction immediately. After drying, the plate was analyzed using an AID plate reader (Autoimmun Diagnostika, Strassberg, Germany).

### Intracellular cytokine staining

Splenocytes from peptide immunized mice were incubated with 1 μM peptide for 1 h [19]. Next, Brefeldin A (Sigma) was diluted 1:1000 and was added for overnight stimulation. The cells were harvested and washed with cold staining buffer. Staining buffer is composed of HBSS (Gibco) and 3 % FCS [27]. The cells were blocked with anti-Fc (BD) for 30 min on ice, followed by a wash with FACS buffer. The cells were stained for CD8 FITC (BD) for 1 h on ice and then washed twice with FACS buffer. Next, the cells were fixed with BD Cytofix for 20 min at RT. The cells were either kept at 4 °C or processed for intracellular staining. To stain, the cells were re-suspended in permeabilization buffer (BD) and washed once with FACS buffer. The cells were then stained for 1 h with PE labeled anti-IFN-γ (BD). Following staining, the cells were washed twice with the permeabilization buffer and re-suspended in FACS buffer. The cells were analyzed by flow cytometry in a FACS Canto using DIVA software.

### Cleaved active caspase-3 and flow cytometry based CTL assay

Splenocytes were cultured with 1 μM of the appropriate peptide for 5 days in culture media supplemented with 10 % T-stim (BD). The cultures were harvested and the cells were centrifuged on Lympholyte M cell separation media (Cedarlane Laboratories, Burlington, NC), washed once in media and adjusted to 10^6^ cells/ml.

K^D^ TAP deficient RMAS cells (a generous gift from Eric G. Pamer, Memorial Sloan Kettering Cancer Center), were maintained in RPMI-10. K^D^ cells were incubated overnight with 1 μM of the appropriate peptide. K^D^ cells were harvested, adjusted to 5 × 10^6^ cells/ml and labeled with 0.6 μM 1,3-dichloro-7-hydroxy-9,9-dimethyl-2(9H)-Acridinone (DDAO; Invitrogen, Grand Island, NY) for 15 min at 37 °C. The reaction was stopped with the addition of complete media. The K^D^ cells were washed once with RPMI-10 and adjusted to 10^6^ cells/ml.

For the Caspase 3 Assay, DDAO labeled peptide pulsed K^D^ cells, 10^5^ in 100 μl of RPMI-10, known as “targets” (T) were added to a round bottom plate [21]. Next, “effector” (E) cells, splenocytes, were added. The E: T ratio was varied from 1:1 to 0.1:1. The plate was spun at 200 rpm for 1 min and the cultures were incubated for 4 h at 37 °C. The cells were washed twice with FACS buffer. The cells were then permeabilized using the cytofix/cytoperm kit according to the manufacturer’s instruction (BD Biosciences) and stained with PE labeled rabbit-anti mouse cleaved Caspase 3 (CC3) at 1:100 dilution for 60 min (BD Biosciences). Cells were analyzed on a FACS Canto using FACS DIVA software.

### Peptide stabilization of MHC

K^D^ cells were washed twice in serum free AIM V media (Gibco), adjusted to 2 × 10^6^ cells per ml and 80 μL was dispensed into each well of a round bottom plate. The peptides, 50 μM, were added to the plate [16]. The plate was incubated for 18 h and then washed with FACS buffer. FITC labeled BB7.2 antibody (BD), diluted to 500 ng/ml, was added for 30 min. After washing the cells were analyzed via FACSCAN.

### Tetramer staining and FACS ARIA sorting

Splenocytes, (10 × 10^6^), from immunized animals were re-suspended in 200 μl sort buffer which contained 2 % FCS and 0.2 % Na azide (Sigma) in HBSS (FACS buffer). Fc receptors were blocked with the addition of anti-Fc (BD) for 20 min. Next, 2 μl of peptide specific PE-labeled tetramer (NIAID Tetramer facility, Atlanta, GA) was added and the cells were incubated for 1 h at 37 °C. After one wash with FACS buffer, the cells were stained with anti-CD3 FITC and CD8 PerCp. Following washing, the cells were fixed as described above and were sorted on a FACS ARIA with 10^6^ events acquired.

### Statistics

All values are expressed as mean ± SD. Individual experiments were performed in at least triplicate (*n* ≥3). The software package Instat 2 (Graph Pad Prism, version 5; Graph Pad Software, San Diego, CA) was used for statistical analysis of data. Significant differences between groups were determined using student’s t-test or one-way analysis of variance (one-way ANOVA) followed by using either a Dunnett’s or Bonferroni’s post-hoc test to compare the significance of differences between means. A *p*-value <0.05 was considered significant.
